# Popular and Scientific Discourse on Autism: Representational Cross-Cultural Analysis of Epistemic Communities to Inform Policy and Practice

**DOI:** 10.2196/32912

**Published:** 2022-06-15

**Authors:** Christophe Gauld, Julien Maquet, Jean-Arthur Micoulaud-Franchi, Guillaume Dumas

**Affiliations:** 1 Department of Child Psychiatry Université de Lyon Lyon France; 2 Department of Internal Medicine Toulouse University Toulouse France; 3 Department of Sleep Medicine Bordeaux University Bordeaux France; 4 Center for Complex Systems and Brain Sciences Florida Atlantic University Boca Raton, FL United States; 5 Center of Research Centre Hospitalier Universitaire Sainte Justine Montréal, QC Canada

**Keywords:** autism spectrum disorder, Twitter, natural language processing, network analysis, popular understanding of illness, knowledge translation, autism, tweets, psychiatry, text mining

## Abstract

**Background:**

Social media provide a window onto the circulation of ideas in everyday folk psychiatry, revealing the themes and issues discussed both by the public and by various scientific communities.

**Objective:**

This study explores the trends in health information about autism spectrum disorder within popular and scientific communities through the systematic semantic exploration of big data gathered from Twitter and PubMed.

**Methods:**

First, we performed a natural language processing by text-mining analysis and with unsupervised (machine learning) topic modeling on a sample of the last 10,000 tweets in English posted with the term #autism (January 2021). We built a network of words to visualize the main dimensions representing these data. Second, we performed precisely the same analysis with all the articles using the term “autism” in PubMed without time restriction. Lastly, we compared the results of the 2 databases.

**Results:**

We retrieved 121,556 terms related to autism in 10,000 tweets and 5.7x109 terms in 57,121 biomedical scientific articles. The 4 main dimensions extracted from Twitter were as follows: integration and social support, understanding and mental health, child welfare, and daily challenges and difficulties. The 4 main dimensions extracted from PubMed were as follows: diagnostic and skills, research challenges, clinical and therapeutical challenges, and neuropsychology and behavior.

**Conclusions:**

This study provides the first systematic and rigorous comparison between 2 corpora of interests, in terms of lay representations and scientific research, regarding the significant increase in information available on autism spectrum disorder and of the difficulty to connect fragments of knowledge from the general population. The results suggest a clear distinction between the focus of topics used in the social media and that of scientific communities. This distinction highlights the importance of knowledge mobilization and exchange to better align research priorities with personal concerns and to address dimensions of well-being, adaptation, and resilience. Health care professionals and researchers can use these dimensions as a framework in their consultations to engage in discussions on issues that matter to beneficiaries and develop clinical approaches and research policies in line with these interests. Finally, our study can inform policy makers on the health and social needs and concerns of individuals with autism and their caregivers, especially to define health indicators based on important issues for beneficiaries.

## Introduction

Autism is characterized in the Diagnostic and Statistical Manual of Mental Disorders, Fifth Edition by the following core deficits: impairments in social interaction and communication as well as restricted, repetitive behaviors [1]. In the 2010 Global Burden of Disease study, an estimated 52 million people had autism worldwide, equating to a prevalence of 1 in 132 individuals [2]. Autism spectrum disorder (ASD) is a neurodevelopmental condition associated with significant health costs, including medical and health care–related service costs, therapeutic costs, education costs, costs of production loss for adults with ASD, costs of informal care and lost productivity for family or caregivers, and costs of accommodation, respite care, and out-of-pocket expenses. The lifetime cost of supporting an individual with ASD is about US $2.4 million, for an individual with ASD and intellectual disability and US $1.4 million for an individual with ASD without intellectual disability [3]. Support service could considerably reduce these costs, but this depends on understanding popular representations of the condition.

**Figure 1:**
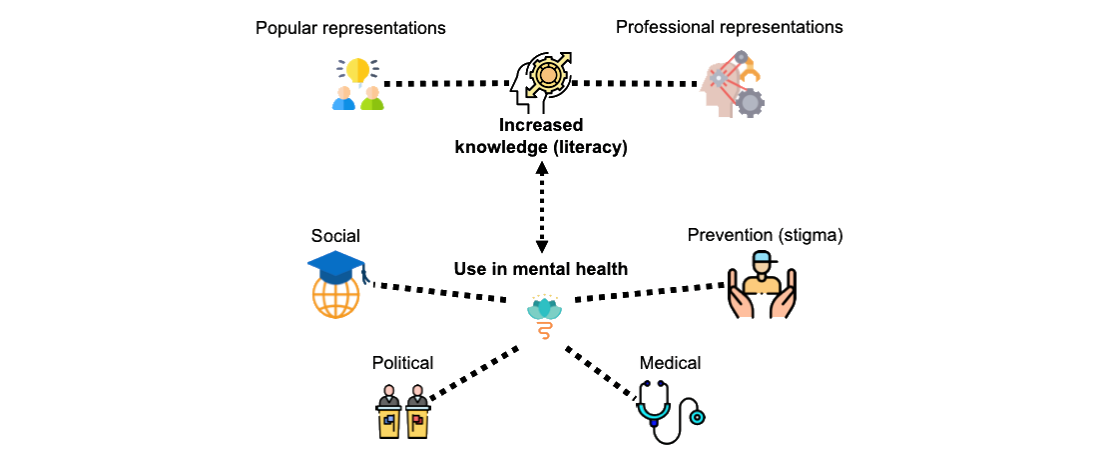
Collaborative learner model between laypeople and health professionals for the improvement of knowledge about mental health (and literacy).

Understanding these lay representations is essential for at least 3 reasons; first, citizens’ decisions can influence research and care policies; second, social, political, and medical attitudes toward mental health recipients are motivated by beliefs about the nature of ASD, especially because lay representations interfere with the understanding of diagnosis and treatment [4]; and finally, popular concepts of mental health have their own logic and implications, are not simply pale reflections of professional concepts filtered through the media, and hence are not shallow, incomplete, and outdated [5]. In fact, there may be significant differences between the perspectives of beneficiaries and researchers. While researchers focus on uncovering underlying mechanisms, beneficiaries and their families may be more concerned with interventions that can immediately improve quality of life. Thus, it becomes critical that mental health professionals and decision makers should be aware of popular representations, especially for high-profile topics such as ASD. Studies on “mental health literacy” are evidence of this lack of overlap between expert and popular concepts [6]. An increase in mental health literacy can have a direct impact on the appropriate use of mental health services [7]. To summarize, popular representations of ASD should be taken into account for the purpose of destigmatization, prevention, education, addressing cultural differences, and developing effective health policies [8]. Figure 1 presents a model of cooperation between lay and expert representations that allows the increase in knowledge on these topics.

Methods for exploring popular representations of a disorder or mental condition are evolving rapidly [9]. Social media provide one way to gain access to popular representations of ASD. As social media have become ubiquitous in everyday life, they represent an easily accessible source of large data sets that reflect popular representations on a wide range of health topics [10]. The aggregation of data from social media can provide insights into first-person experience and daily life engagements with various health conditions.

More specifically, Twitter is the most widely used social media in public health and is considered the “Internet radio.” An estimated 3.5 million users visit Twitter each month [11], with 336 million monthly active users and 500 million tweets sent per day and a high global adoption rate (77% of its users are located outside the United States). In addition, while most social media data (eg, Facebook) remain private, all Twitter’s data are publicly available. A significant portion of Twitter’s messages focus on health-related topics [12], and beneficiaries are increasingly turning to it to keep abreast of health developments and better understand their condition [11,12]. Twitter can thus potentially serve as a large-scale interactive platform to reach ASD-affected communities that may be difficult to reach through traditional means.

A brief search of the literature on ASD using the term “mining” shows that only 60 articles have been published on this subject, and that most concern the genetics of autism (19/60, 32%). To our knowledge, the most comprehensive literature review of textual analysis of autism on social media identified 5 articles [13-17]. This study is the first to analyze lay representations of autism and compare them with those of the scientific community.

## Methods

We compared the representations of autism in the social media and the scientific literature using the following three steps: (1) textual analysis of data from Twitter; (2) textual analysis of articles on PubMed; and (3) the comparison of these two corpora to highlight the differences between lay representations (by proxy via Twitter) and scientific representations (by proxy via PubMed).

### Textual Analysis of Twitter

In the first phase, we conducted an extraction and a textual analysis of mentions of autism on Twitter on January 21, 2021, using a sample of the last 10,000 tweets in English, posted using the term “#autism.” We did not apply any geographical limits. We made sure to choose a random day, and especially to avoid any particular day of the year or a specific day related to autism (eg, the entire month of April, aka the “autism month”). This number of tweets (N=10,000) constitutes the maximum number of tweets that can be retrieved by the application programming interface (API) of this platform. All data were obtained through the official Twitter API. We did not use the term “autis*” in order to specifically target tweets related to a theme centered mainly on autism. The term “#autism” can better identify tweets that target a specific message on autism, because the hashtag can help to avoid tweets in which the theme of autism is only secondary (the “hashtag” [#] groups together all the discussions referring to the same theme). No other apparently irrelevant term was deleted, assuming that the relevance of such an analysis lies in reading the topics considered as patterns and not at the level of over-selected results. We extracted the terms most commonly associated with autism using the R software (version 4.0.3, The R Foundation; packages: stringr, rtweet, tidyr, tidytext, rjson, and leaflet) and analyzed the resulting big data on the Béluga supercomputer (*Compute Canada*) in 4 steps.

First, we prepared the data (data munging) by removing the URLs and hyperlinks (high-frequency words) by applying the standard techniques of preprocessing data for text analysis, which comprises (1) punctuation marks, (2) numeric characters, (3) spaces, and (4) special symbols. We associated a unique identifier with each occurrence of a word. Stop words, the most common words in a language that have no interest in the analysis (eg, “and,” and “for”) were also removed (tidytext package).

Secondly, we carried out a bootstrap analysis with an algorithm specifically created for the study, to analyze words and not tweets, in order to extract the first terms and test the network’s stability (NetworkTools package).

Thirdly, we performed an undirected lexical network analysis (qgraph package) to map these terms and dimensions in space and observe their relationships, following the network analysis guidelines for cross-sectional data published by Burger et al [18] (Multimedia Appendix 1). We extracted the 50 main common words to build this network. Thus, the lexical network was constructed by Twitter mining with the term #autism with the first 50 terms. Such a lexical network can be considered as undirected because it does not describe a causal or directional relationship between the nodes. A connection between any 2 terms is represented if these terms are frequently present together in a tweet or an article. In network analysis, the potential importance of nodes within the network can be assessed by the following 4 local measures of network centrality: *strength*, *closeness*, *betweenness* (interval between nodes), and *expected influence* [19]. The *strength* of a node measures the weighted number of connections for a given node, thus showing the degree of involvement of this node in the network. *Closeness* is inversely proportional to the shortest average distance to all other nodes. *Betweenness* measures the degree to which a given node acts as a “bridge” connecting different parts of the network, reflecting the degree by which it controls the flow of information across the network. *Expected influence* is computed to improve the measurement of the centrality of nodes in the network, reflecting the influence on the symptom network based on its positive correlations, negative correlations being corrected by this centrality measure.

Finally, we used an unsupervised machine learning technique capable of scanning tweets to detect word patterns and to automatically cluster word groups, with an algorithm capable of determining the ideal number of clusters, based on the computation of a dissimilarity matrix with Euclidean distance and a cluster analysis by a k-means method (k-means reallocation clustering minimizes within-cluster variances, that is, squared Euclidean distances; it minimizes the sum of distances between the points and their respective cluster centroid; NbClust package). The result is given in the form of a numerical clustering index; in this study, the index used is the C-index: the higher the C-index, the more relevant the number of clusters. This cluster analysis provides a representation of the main semantic dimensions. More specifically, the cluster analysis is based on a method entitled Latent Dirichlet Allocation (LDavis package), which is a generative statistical model explaining sets of observations through unobserved groups, which are themselves defined by data similarities (topicmodels package). The dimensions extracted according by Latent Dirichlet Allocation correspond to the terms that frequently occur together, based on how frequently the word is on an exact topic. This cluster analysis is thus called topic modeling. Each of these topics is labeled on expert opinion according to the lexical fields of the terms found in the clusters. Successive blind iterations were performed until a common agreement was found between the authors. The labels are not methodologically formalized, but they are given according to the terms extracted and presented in the figure. Each of these labels, interpreted qualitatively, should be read according to the sets of terms of the figure.

### Textual Analysis of PubMed

In the second phase, we performed an extraction and a textual analysis of mentions of autism using the biomedical database PubMed, in January 2021 (the same day as Twitter’s collection of terms), using the term “autis*” (Multimedia Appendix 1). The analysis of terms included titles, keywords (including MeSH [Medical Subject Headings]), and abstracts. We used for the PubMed analysis the same 4 steps procedure as for the Twitter database (data preparation, bootstrap analysis, undirected lexical network analysis, and clustering with topic modeling).

### Comparison of Corpora

The comparison between the 2 corpora has been carried out qualitatively. Despite the absence of the possibility of developing quantitative analyses due to the intrinsically different labels of the networks, the development of similar analysis tools for each database (associated with the parallel extraction of dimensions) will allow room for interpretation in the Discussion section.

### Ethical Considerations

Internet-related research raises specific ethical considerations as to whether the obtained data belong to the public or private domain [20], with respect for confidentiality and valid consent [21]. The handles were not kept in the results and therefore all the data remain at the level of the statistical aggregate. Only publicly available data on the web and collected from the Twitter platform were analyzed. We only used data from anonymous users who consented to publicly disclose their data on Twitter (ie, no privacy settings were selected by users; Multimedia Appendix 1).

## Results

### Data Mining and Bootstrapping

#### Textual Analysis of Twitter

The request was launched in January 2021, searching for 10,000 unique tweets (not retweeted) using the term “#autism.” We retrieved 121,556 terms related to the term #autism. All these tweets (not retweeted) were posted in English on Twitter in less than a month, without geographical limits.

#### Textual Analysis of PubMed

In January 2021, the PubMed literature search identified 57,121 articles with the term “autis*” and with 5.7x10^9^ terms. The distribution of items over time (timeline) shows an average of 654 items per year smoothed over the last 70 years, with the first articles published in 1946.

**Figure 2:**
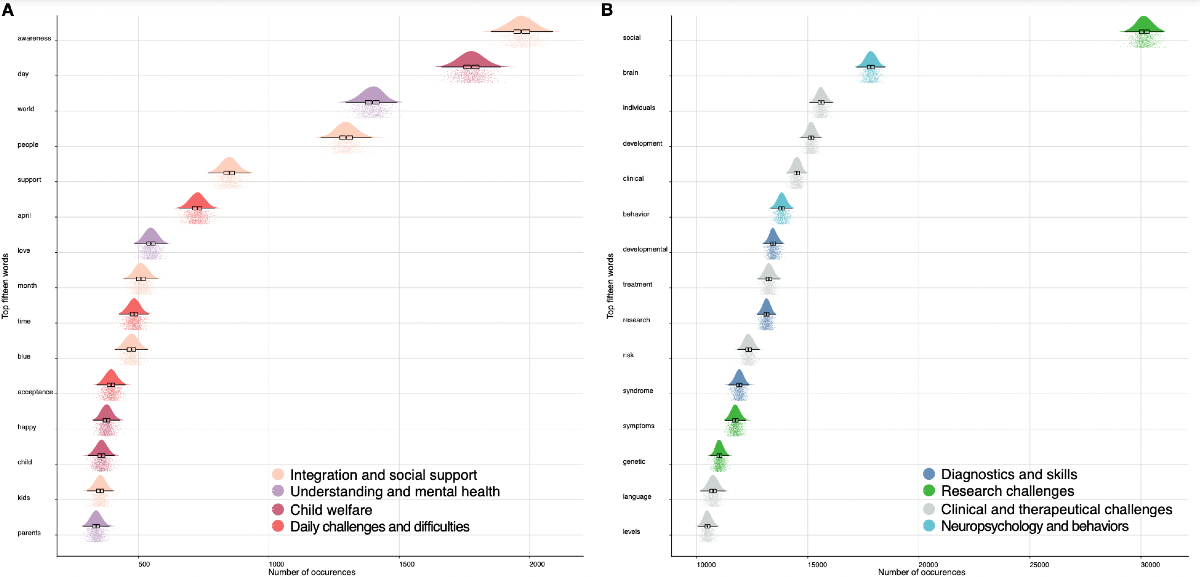
A. 15 first terms found on Twitter by textual analysis on 10,000 tweets in paired analysis using the term #autism (January 2021). The term “autism” itself has been removed from the list for better visibility. B. 15 first terms found on PubMed by textual analysis on 57,121 articles using the term “autis*” (January 2021). The term “autism” itself has been removed from the list for better visibility.

The bootstrap algorithm (allowing to analyze words and not tweets) extracts the most frequently used terms and allows to test the network’s stability. The confidence intervals (frequency of terms in tweets) of the bootstrap are narrow. Figure 2 shows the 16 terms found most frequently associated with the searched keyword on Twitter (panel A) and PubMed (panel B). The networks were stable at 1000 iterations, as shown by the bootstrap analyses.

Based on globally equivalent distributions, the frequency of the first 15 terms found in the PubMed database is between 10,000 and 18,000 times (ie, a frequency of about 2.5x10^-4^). The frequency of the first 15 terms found in the Twitter database is between 250 and 2000 times (ie, a frequency of about 0.92%) with different distributions (Figure 2).

### Undirected Lexical Network Analyses

**Figure 3:**
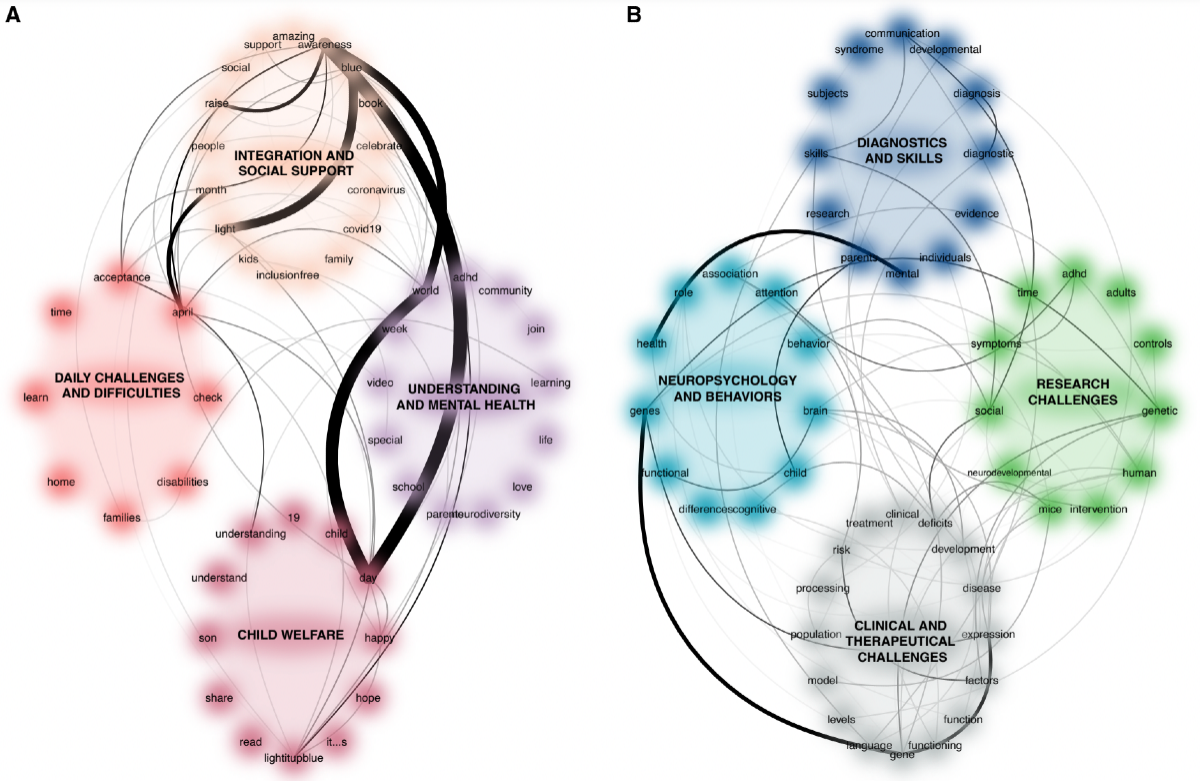
A. Directed network of 50 first words from Twitter with #autism after textual analysis of 10,000 tweets. B. Directed network of 50 first words from PubMed with “autis*” after textual analysis of all the articles on the database.

The first lexical network was constructed by Twitter mining using the term #autism with the first 50 terms among the 121,556 terms in the 10,000 unique tweets. The second lexical network was constructed by PubMed mining with the term “autis*” with the first 50 terms (among the 5.7x10^9^ terms in the 57,121 articles). Figure 3 shows these undirected lexical network analyses. Remember that a connection between any 2 terms is represented if these terms are frequently present together in a tweet or an article.

The qualitative analysis of these networks shows marked differences between the terms associated with autism on Twitter and those in PubMed. For example, on Twitter, we found terms such as “Son,” “Love,” “Happy,” or “April” (referring to the World Autism Month) denoting special community attention to the idea of well-being in ASD, but also others such as “Neurodiversity,” “ADHD” (for attention deficit hyperactivity disorder), and “Brain” focusing attention on the scientific aspects of ASD. Moreover, terms such as “Acceptance,” “Social,” or “Understanding” were related. Among the most common terms in the literature, the PubMed analysis revealed words belonging to various scientific lexical fields such as “Gene,” “Brain,” or “Cognitive,” denoting a special scientific focus on physiopathological mechanisms, similar to the terms “Communication,” “Skills,” or “Social,” denoting special focus on the functional issues involved in ASD.

**Figure 4:**
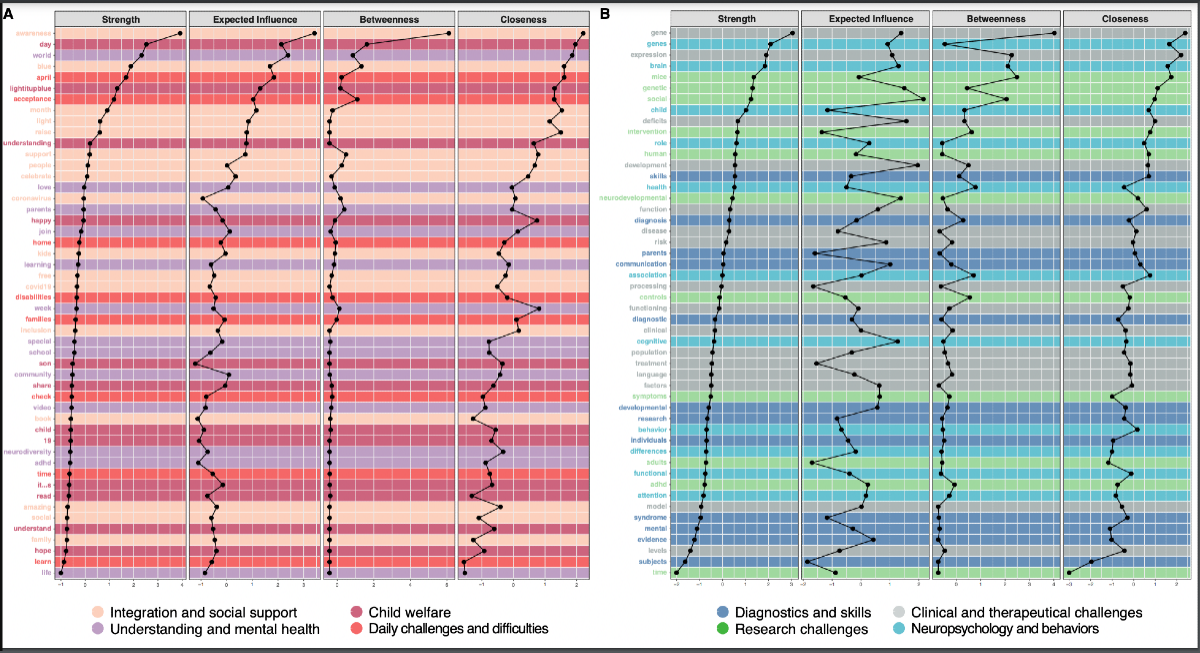
Four centrality measures (strength, expected influence, betweenness, and closeness), ordered by expected influence of A. Twitter network and its 4 dimensions; B. PubMed network and its 4 dimensions (details of the different centrality measures are in the Methods section). The most central node in terms of strength, for each database, is located at the top of each of the tables (eg, node “Awareness” for the Twitter network and node “Gene” for the PubMed network). A high centrality node is located to the right of each table, and a low centrality node is located to the left.

The centrality measures of these networks (Figure 4) showed that in Twitter, the 5 most central terms related to strength, expected influence, and closeness were as follows: “Maths,” “Goals,” and “Stem.” In terms of betweenness, they were related to “Students,” “Maths,” and “Disabilities.” In PubMed, the 3 most central terms related to expected influence were as follows: “Neurodevelopment,” “Attention,” and “Deficit.” In terms of strength, they were “Expression,” “Gene,” and “Brain.” In terms of betweenness, they were also “Expression,” “Gene,” and “Social.” In terms of closeness, they were also “Expression,” “Gene,” and to “Development.”

### Topic Modeling to Extract Main Semantic Dimensions

We determined the number of clusters and identified the best clustering scheme by varying all combinations of the number of clusters, distance measures, and clustering methods. For both Twitter and PubMed, the best clustering schematization algorithm (based on the Euclidean distances and k-mean clustering) proposed 2 dimensions with a C-index of 235.4, and 4 dimensions with a lower C-index of 183.7. We retained this quantitative result but decided to use 4 dimensions for both networks for semantic reasons. After iterative tests with values greater than 4, it seemed more appropriate to use 4 dimensions to interpret and compare the 2 networks, and to compare the 2 corpora more closely. This approach is clinically relevant, since only 2 isolated dimensions would not be sufficiently informative in a study comparing different dimensions within different corpora. The 4 main dimensions extracted are shown in Figure 3. The dimensions in the Twitter network were as follows: (1) integration and social support; (2) understanding and mental health; (3) child welfare; and (4) daily challenges and difficulties. The dimensions in PubMed were as follows: (1) diagnosis and skills; (2) research challenges; (3) clinical and therapeutical challenges; and (4) neuropsychology and behavior.

## Discussion

### Principal Results

We produced 2 corpora of texts from web-based platforms representing a global social network (Twitter) and international biomedical research (PubMed), reflecting both the interests and representations of the scientific community and mental health professionals. The content of the tweets and scientific articles were explored by searching within these samples for the terms #autism and autis*, respectively. We found a wide divergence in the focus of these corpora, which has implications for how researchers and the public at large understand each other’s discourse.

The 4 dimensions extracted from Twitter (integration and social support, understanding and mental health, child welfare, and daily challenges and difficulties) reveal a discourse focusing on the education, evolution, and support for people with ASD that makes relatively little mention of science or technical issues per se. Likewise, the 4 dimensions extracted from PubMed (diagnosis and skills, research challenges, clinical and therapeutical challenges, and neuropsychology and behavior) reflect scientific research practices and reveal an interest in behavioral and linguistic issues, comorbidities ,and neuroscientific topics (eg, neuropsychology, neurogenetics, and neuropharmacology), with very little mention of the issues dear to Twitter users.

Since several scientific communities are involved, the construct of ASD has been fraught with controversy [22,23]. ASD is a multiscale condition, and its scientific study requires different levels of analysis and generates various points of view, with each community providing its own perspective. These specialists include neuroscientists, biologists, pharmaceutical engineers, clinical researchers, and practitioners. Lay communities interested in ASD include parents, parents’ and beneficiaries’ associations, and associations that support beneficiaries daily. The topics commonly discussed by the scientific community did not appear to overlap with the concerns of the public. This lack of overlap has 3 important implications for research. First, from a scientific perspective, the range of topics covered by Twitter could provide health care professionals and researchers with insights that they would otherwise not have (Figure 1). Second, from an economic perspective, the study of social media can increase health professional’s knowledge of public and patient concerns at low cost, given the relatively free access to personal data that people disseminate about themselves. Third, in terms of public health and interventions, gaining such knowledge of the centers of interest and concerns of the public and patients could help them target social media communities involved with ASD in terms of information and interventions, all at a minute cost. Indeed, the terms found in tweets could reveal trends in the representation of mental disorders or conditions. This would help professionals provide appropriate public health information, set up prevention campaigns, destigmatize the ASD condition, and engage decision-making through an indirect suggestion for significantly changing their representational incentives. In consequence, such divergences may be useful for scientific dynamics, public health information and prevention efforts, psychoeducation, or management of care.

While the representations of ASD found in popular discourse and in biomedicine differ, they do not diverge completely. In fact, the views of professionals regarding psychiatric disorder and conditions, comorbidities, and their implications for families have a considerable influence on social media discussions. However, the differences of focus in the medical literature and the general population are important, which involves implications in terms of naturalization of a disorder and overmedicalization of a condition. In popular representations and therefore in social media, these representations may provoke negative attitudes, encouraging people to consider such a condition as deeply ingrained or constitutional and categorically different from people considered neurotypical [24]; they may also increase stigmatization or increases perceptions of dangerousness and unpredictability about patients [25]. Certainly, ASD poses a particularly challenging issue since laypeople may consider that those with ASD are biologically but not pathologically different, particularly in the context of neurodiversity [26]. However, a uniquely medical understanding of a condition such as ASD may lead to the conviction that those with ASD are unable to function normally, a belief that can lead to pessimism and disengagement if it is held widely by the public [27].

### Limitations

This study has several limitations, 5 of which will be discussed here. First, for methodological reasons related to text processing, we limited the searches to the English language and to 2 databases, although both produced large corpora representative of very heterogenous populations. Indeed, texts on social media are known to contain a large proportion of nongrammatical constructions derived from abbreviations and metaphorical uses [28], while scientific texts are known for their specialized vocabulary and characteristic structure that make them a genre per se. Thus, automated comparison of the 2 requires careful annotation. On the other hand, automated methods allow large corpora to be explored, a task that would indeed be challenging if it were to be performed by close textual analysis. Furthermore, they can reveal patterns despite the existence of significant differences in the structure of discourse from various origins [29]. But even if it is possible that the 2 corpora (Twitter and PubMed) do not simply represent 2 different populations or perspectives perfectly distinctive (and are only 2 different modes of communication), this does not change the scope of our results because the claims about differences in concerns regarding the 2 corpora remains; the themes are different in terms of interests. However, we do not wish to exclude individual representations conveyed by the health professionals, who are a significant part of the social landscape.

Second, in our study, first-person representations of individuals with ASD active on Twitter were not considered. This could have been carried out by combining the terms “#autism” and “I.” A recent report noted that about 80% of adults living with ASD use social media. Examination of first-person representations would provide a valuable avenue for future research on phenomenology and the mediatized social presentation of self [13]. Third, the use of isolated words and not n-grams (contiguous sequence of terms) could constitute a future perspective, since it would be a question of analyzing the relevance of pertinent contiguous occurrences in ASD (eg, the term “gene” does not have the same meaning if it is associated with “expression” or with “deficit,” providing potential important information on the genetics of ASD).

Fourth, this study has the limitations of any study based on social networks and textual data mining, including the sampling bias related to users of this medium (eg, in terms of social class or culture), and the difficulty of checking user profiles [15], search parameters, or the necessary qualitative labeling of clusters. In particular, the use of the hashtag (“#autism”) cannot provide the same precision as the MeSH term (“autis*”), with a potential number of false-positive tweets (ie, not specifically related to ASD). In addition, concerning the PubMed corpus, we may have included articles with the term “autis*” in the abstract but not related to autism, even though this eventuality was potentially rare.

Fifth, for computational challenges, we had to limit the number of calculations, so we were unable to perform a series of data extractions. Indeed, the Twitter API limits data extraction for ethical reasons. However, the limitations related to technological access and data processing were offset by the rewriting of specific algorithms for the purpose of this study and by the amount of data that we acquired. The narrowness of the confidence intervals of the frequency of terms obtained by bootstrapping confirms the accuracy and robustness of our data estimation. These limitations are also partially offset by the reduction in social desirability and recall bias compared to traditional survey data collection methods. This observation calls for future studies integrating a variety of data from different sources.

### Conclusion

Apart from the stigma that ASD induces, the exposure of ASD also leads to suffering in relatives of people with ASD, who feel a sense of social injustice in that clinical research is still unable to meet their expectations for their loved ones. This paper illustrates the potential to use social media as a proxy for the representations of ASD in the society today. The results suggest a clear distinction between the focus of topics used in the social media and that of scientific communities. This highlights the importance of knowledge mobilization and exchange to better align research priorities with personal concerns and to address dimensions of well-being, adaptation, and resilience. Similar methods could be used to develop pedagogical or preventive programs, or to help in establishing recommendations for treatment. The analysis of representations prevalent in the social media could also be used to design destigmatization campaigns and to assess their impact over time.

As noted by Hacking [30], such representations strongly impact not only care but also research and nosology. The interaction between shared representations and medical research interests could help public health decision makers and mental health professionals to create collaborative learning environments that engage beneficiaries and caregivers.

## Appendix

Multimedia Appendix 1 Supplementary files.

## Abbreviations

ADHD: attention deficit hyperactivity disorder
API: application programming interface
ASD: autism spectrum disorder
MeSH: Medical Subject Headings
