# Supplementary material for: Popular and Scientific Discourse on Autism: Representational Cross-Cultural Analysis of Epistemic Communities to Inform Policy and Practice
Source: J Med Internet Res. 2022 Jun 15;24(6):e32912. doi: 10.2196/32912 (PMC9244652; doi:10.2196/32912)
Supplement: Multimedia Appendix 1 [file jmir_v24i6e32912_app1.docx]

**Multimedia Appendix 1. Supplementary Material**

***Research on PubMed (autis*)***

(autis[All Fields] OR autischen[All Fields] OR autischer[All Fields] OR autisense[All Fields] OR autisic[All Fields] OR autisiclike[All Fields] OR autisim[All Fields] OR autisitc[All Fields] OR autisitcsubjects[All Fields] OR autism[All Fields] OR autism'[All Fields] OR autism's[All Fields] OR autism1[All Fields] OR autism360[All Fields] OR autismaid1[All Fields] OR autismand[All Fields] OR autismate[All Fields] OR autismcare[All Fields] OR autismcenter[All Fields] OR autismcrc[All Fields] OR autismdata[All Fields] OR autisme[All Fields] OR autisme'[All Fields] OR autismeexpertise[All Fields] OR autismeforeningen[All Fields] OR autismen[All Fields] OR autismes[All Fields] OR autismespectrum[All Fields] OR autismespectrumstoornis[All Fields] OR autismespectrumstoornissen[All Fields] OR autismespekterforstyrrelser[All Fields] OR autismeteam[All Fields] OR autismeteamet[All Fields] OR autismevriendelijke[All Fields] OR autismi[All Fields] OR autismikirjon[All Fields] OR autismiliitto[All Fields] OR autismkb[All Fields] OR autismlike[All Fields] OR autismmatch[All Fields] OR autismmedicalhome[All Fields] OR autismo[All Fields] OR autismor[All Fields] OR autismos[All Fields] OR autismpro[All Fields] OR autismqld[All Fields] OR autismresearch[All Fields] OR autisms[All Fields] OR autisms'[All Fields] OR autismselfadvocacy[All Fields] OR autismspeaks[All Fields] OR autismspectrum[All Fields] OR autismspektrumbegreppet[All Fields] OR autismspektrumstorning[All Fields] OR autismspektrumsyndrom[All Fields] OR autismspektrumtillstand[All Fields] OR autismstudie[All Fields] OR autismstudies1[All Fields] OR autismstudiesnet[All Fields] OR autismstudynurseasu[All Fields] OR autismtherapies[All Fields] OR autismu[All Fields] OR autismul[All Fields] OR autismului[All Fields] OR autismus[All Fields] OR autismusbehandlung[All Fields] OR autismusforschung[All Fields] OR autismusspektrumstorungen[All Fields] OR autismussyndrom[All Fields] OR autismustherapiezentrum[All Fields] OR autismuszentrum[All Fields] OR autisn[All Fields] OR autisru[All Fields] OR autissier[All Fields] OR autissiodorensis[All Fields] OR autist[All Fields] OR autista[All Fields] OR autistacon[All Fields] OR autistas[All Fields] OR autiste[All Fields] OR autisten[All Fields] OR autistes[All Fields] OR autisti[All Fields] OR autistic[All Fields] OR autistic'[All Fields] OR autistic's[All Fields] OR autistica[All Fields] OR autistical[All Fields] OR autistically[All Fields] OR autisticand[All Fields] OR autisticas[All Fields] OR autisticheskii[All Fields] OR autisticheskikh[All Fields] OR autisticheskogo[All Fields] OR autisticheskoi[All Fields] OR autisticheskom[All Fields] OR autistici[All Fields] OR autistickeho[All Fields] OR autistickych[All Fields] OR autisticlike[All Fields] OR autisticne[All Fields] OR autisticnog[All Fields] OR autisticnom[All Fields] OR autistico[All Fields] OR autisticos[All Fields] OR autistics[All Fields] OR autistics'[All Fields] OR autistiform[All Fields] OR autistique[All Fields] OR autistiques[All Fields] OR autistisch[All Fields] OR autistische[All Fields] OR autistischem[All Fields] OR autistischen[All Fields] OR autistischer[All Fields] OR autistisches[All Fields] OR autistischundisziplinierten[All Fields] OR autistisk[All Fields] OR autistiska[All Fields] OR autistiske[All Fields] OR autistism[All Fields] OR autististic[All Fields] OR autistm[All Fields] OR autistoid[All Fields] OR autists[All Fields] OR autisyn[All Fields]) AND ("diagnosis"[MeSH Terms] OR "diagnosis"[All Fields] OR "diagnostic"[All Fields]) AND Review[ptyp]

***Ethics***

Internet-related research raises specific ethical considerations as to whether the obtained data belong to the public or private domain[1], with respect for confidentiality and valid consent [2,3]. By following the work of Hswen, Gopaluni, Brownstein and Hawkins [4] on the textual analysis of autism and those of Suominen and colleagues [2] and Charlesworth and Tonkin [5] on the ethics of textual analysis this study was considered exempt from ethical approval. Indeed, only publicly available data on the Web and collected from the Twitter platform were analyzed^[[1]](#footnote-1)^. To ensure the confidentiality of the data in this group, the collected data were aggregated and analyzed at the population level and anonymized in this publication format. In addition, this study only used data from users who consented to publicly disclose their data on Twitter (no privacy settings were selected by users). The handles are not kept in the results and therefore all the data remains at the level of the statistical aggregate. As a guarantee of reproducibility, we have added the codes Data and code to generate the figures are available at:

https://github.com/ChristopheGauld/TwiMed.

***Network Analysis***

The main aim of a textual network analysis is to analyze the units of analysis (i.e. terms extracted from the database) described in a database. In this methodology, the correlations between variables (i.e. terms or units of analysis extracted from the database) can be understood as a network in which each variable is a unit of analysis representing a “node”, and where correlations between variables are “connections” (or “edges”) between nodes. A connection between any two terms is represented if these terms are frequently present together (according to a measure of Euclidian distance) in a tweet or an article. The network used in this study is called “undirected” to indicate the absence of directional relationship between the nodes in the network. A network representing all units of analysis and connections between them had to be constructed graphically (network estimation), quantified with classical metrics (network inference with global measures, i.e. clustering coefficient and average shortest path length and local measures, i.e. centrality measures) and tested for robustness (network robustness) [6], following the network guidelines for the computational analysis of network properties [7].

*Network estimation*

A main advantage of the network approach is that it visualizes the multivariate dependencies of the data that otherwise remain hidden, especially on such big data for this study. The most informative visualization is one in which edges can be understood as partial correlation coefficients, meaning that a connection between nodes is the connection after controlling for all other edges in the network [8].

Based on a correlation matrix obtained with Pearson correlations (in order to show side by side the association of two terms), the textual network was graphically represented according to a group algorithm. Indeed, in this graphic representation, the place of the node on a map is related to its group.

*Network accuracy*

Network accuracy was conducted with an Ising Model, which is to date the state-of-the-art network model used in psychopathology research for binary data. An Ising model can be understood as estimating partial correlations among a set of binary items. It comprises two important processes: the estimation of pairwise relationships between the symptoms (i.e. the edges) and a network regularization (i.e. to consider the entire network), limiting the number of non-null edges according to sample size. The estimation of pairwise relationships between the symptoms can be seen as a conditional dependence relation. An association between two symptoms means that they remain conditionally dependent after controlling for all other associations among the symptoms in the global network. Conversely, if no edge emerges between two symptoms, they are conditionally independent after controlling for the associations among all other symptoms.

In order to minimize the risk of false positives (i.e., a nonzero connection estimated although there is no actual connection), we use a regularization technique called graphical least absolute shrinkage and selection operator (LASSO) [9]. The LASSO penalizes the sum of absolute parameter values such that the estimated values shrink to zero. A tuning parameter lambda (λ) provides different estimating network structures for various lambda values. The final model is chosen by using the extended Bayesian information criterion (BIC) [10]. The result is a sparse and conservative network, whose connections that are estimated to be present (i.e., nonzero) are likely true positives, whereas the connections that are estimated to be absent (i.e., zero) may be false negatives.

Based on this estimation, a correlation matrix was obtained with Pearson correlations (in order to show side by side the association of two symptoms in the diagnostic criteria of sleep disorders). The symptom network was graphically represented according to a Fruchterman-Reingold algorithm [11]. In this graphic representation, the weight of the connection between two nodes is proportional to the correlation measures, and the place of the node is positioned according to a force-directed graph measure, so that symptoms with stronger and/or more connections are placed closer to each other.

*Network inferences: local measures*

The local measures of a network could be related to centrality measures [12]. Centrality measures are important for identifying bridge variables, which play a crucial role in connecting nodes [13], and can be assessed by using bridge centrality statistics [14–16]. The centrality parameters of a network are particularly important to better understand the relationship between terms in such a textual network. Nodes with high centrality index measures represent variables that are highly connected to other variables. The correlations between variables may be considered as stronger when the nodes have higher centrality. These centrality statistics can be quantified by four measures: Strength, Closeness, Betweenness and Expected Influence.

The strength of a node computes the weighted number of connections for a given node, and therefore the degree to which it is connected with all the other nodes of the network [17]. A variable has high strength centrality if this variable is highly connected to all the other variables. In a rail network metaphor, a city has high strength if it is connected to an extremely large number of other cities, e.g. Paris in France.

The closeness of a node is computed according to the shortest path length measure and is inversely proportional to the shortest mean distance from all the other nodes [12]. A variable has high closeness centrality if the variable can be connected shortly to other variables [18–20]. Using the rail network metaphor, a city has high betweenness if it is central compared to many other cities, i.e. it is “close” to many others, like the city of Bourges which is very central in France.

The betweenness of a node computes the degree to which a given node acts as a “bridge” connecting different parts of the network, thus reflecting the degree to which it controls the flow of information across the network. A variable has high betweenness centrality if the variable can influence the connection between non-adjacent (i.e. not directly connected) variables, thus acting as gatekeeper [19,21]. In the railroad network metaphor, a city has high closeness if it is necessary to transit through that city to reach other cities, like the city of Lyon which must be passed through on the route between Paris and Marseille.

The Expected Influence of a node is computed to improve the measurement of centrality of the nodes in a network [22]. It is computed as the sum of all edges which extend from a given node, accounting for both positive and negative correlation values with regard to the entire network. A variable with high Expected Influence has an influence on the variable network based on its positive correlations, negative correlations being corrected by this centrality measure. In the railroad network metaphor, a city has a high expected influence if it is connected to an extremely large number of connected neighboring cities, considering possible negative correlation values between cities.

*Network robustness*

When estimating textual networks, a major current challenge is that the stability and accuracy of these networks and their centrality measures are unclear [23]. Three steps were conducted to assess the accuracy of variable network structures: i) estimating the confidence intervals on the edge-weights; ii) assessing the stability of centrality indices under observing subsets of cases; iii) and testing for significant differences between edge-weights and centrality indices.

Network robustness (or stability analysis) was calculated by an analysis written by the authors and using a bootstrap method (N= 2,000) based on the association of a resampling and a leave-one-out, with confidence intervals based on bootstrap percentiles and not on standard deviations. If correlation values decline substantially as participants are removed, then this centrality index would be considered less stable. This index is given by the value of the CS-coefficient:  it represents the maximum proportion of participants that can be dropped while maintaining 95% probability that the correlation between centrality metrics from the full data set and the subset data are at least .70. Based on a simulation study [10], a minimum CS-coefficient of 0.25 is recommended for interpreting centrality indices. If correlation values decline substantially as participants are removed, then this centrality index would be considered less stable.

**Computational Analysis**

Processing and graphical visualizations used the R (4.1.0) package bootnet (version 1.2.3) [6], Pearson correlations and with the qgraph package for visualization (version 1.6.3) [24]. Note that the bootnet algorithm was adapted to fit the format of our dataset (i.e. not multiple patients with their variables, but disorders with associated variables). Expected influence values were calculated with the networktools R package. Network robustness was calculated by an analysis rewritten by the authors and using a bootstrap method (N= 2,000) based on the association of a resampling and a leave-one-out. The robustness of the results is tested only according to the disorders and not to the variables, as would have been the case with the bootnet package.

**References**

1. Dumas G, Serfass D, Brown N, Sherman R. The Evolving Nature of Social Network Research: A Commentary to Gleibs (2014). 2014; [doi: 10.1111/ASAP.12055]

2. Suominen H, Lehtikunnas T, Back B, Karsten H, Salakoski T, Salanterä S. Theoretical considerations of ethics in text mining of nursing documents. Stud Health Technol Inform 2006;122:359–364. PMID:17102280

3. Hewson C, Buchanan T. Ethics guidelines for internet-mediated research. The British Psychological Society; 2013.

4. Hswen Y, Gopaluni A, Brownstein JS, Hawkins JB. Using Twitter to Detect Psychological Characteristics of Self-Identified Persons With Autism Spectrum Disorder: A Feasibility Study. JMIR Mhealth Uhealth [Internet] 2019 Feb 12 [cited 2021 Mar 3];7(2). PMID:30747718

5. Charlesworth A, Tonkin EL. Chapter 3 - If You Find Yourself in a Hole, Stop Digging: Legal and Ethical Issues of Text/Data Mining in Research. In: Tonkin EL, Tourte GJL, editors. Working with Text [Internet] Chandos Publishing; 2016 [cited 2021 Mar 3]. p. 61–88. [doi: 10.1016/B978-1-84334-749-1.00003-2]

6. Epskamp S, Borsboom D, Fried EI. Estimating psychological networks and their accuracy: A tutorial paper. Behavior Research Methods 2018 Feb;50(1):195–212. [doi: 10.3758/s13428-017-0862-1]

7. Burger J, Isvoranu A-M, Lunansky G, Haslbeck J, Epskamp S, Hoekstra RHA, Fried EI, Borsboom D, Blanken T. Reporting Standards for Psychological Network Analyses in Cross-sectional Data [Internet]. PsyArXiv; 2020. [doi: 10.31234/osf.io/4y9nz]

8. Armour C, Fried EI, Deserno MK, Tsai J, Pietrzak RH. A network analysis of DSM-5 posttraumatic stress disorder symptoms and correlates in U.S. military veterans. J Anxiety Disord 2017 Jan;45:49–59. PMID:27936411

9. Friedman J, Hastie T, Tibshirani R. Sparse inverse covariance estimation with the graphical LASSO. Biostatistics (Oxford, England) 2008 Aug 1;9:432–41. [doi: 10.1093/biostatistics/kxm045]

10. Epskamp S, Borsboom D, Fried EI. Estimating Psychological Networks and their Accuracy: A Tutorial Paper. arXiv:160408462 [stat] [Internet] 2017 Jan 20 [cited 2020 Oct 6]; Available from: http://arxiv.org/abs/1604.08462

11. Fruchterman TMJ, Reingold EM. Graph drawing by force-directed placement. Software: Practice and Experience 1991 Nov;21(11):1129–1164. [doi: 10.1002/spe.4380211102]

12. Boccaletti S, Latora V, Moreno Y, Chavez M, Hwang D. Complex networks: Structure and dynamics. Physics Reports 2006 Feb;424(4–5):175–308. [doi: 10.1016/j.physrep.2005.10.009]

13. Cramer AOJ, Waldorp LJ, Maas HLJ van der, Borsboom D. Comorbidity: A network perspective. Behavioral and Brain Sciences 2010 Jun;33(2–3):137–150. [doi: 10.1017/S0140525X09991567]

14. Heeren A, Jones PJ, McNally RJ. Mapping network connectivity among symptoms of social anxiety and comorbid depression in people with social anxiety disorder. Journal of Affective Disorders 2018 01;228:75–82. PMID:29232567

15. Jones PJ, Heeren A, McNally RJ. Commentary: A network theory of mental disorders. Frontiers in Psychology [Internet] 2017 [cited 2020 Feb 16];8. [doi: 10.3389/fpsyg.2017.01305]

16. Opsahl T, Agneessens F, Skvoretz J. Node centrality in weighted networks: Generalizing degree and shortest paths. Social Networks 2010 Jul;32(3):245–251. [doi: 10.1016/j.socnet.2010.03.006]

17. Barrat A, Barthélemy M, Vespignani A. Weighted Evolving Networks: Coupling Topology and Weight Dynamics. Physical Review Letters 2004 Jun;92(22):228701. [doi: 10.1103/PhysRevLett.92.228701]

18. Smith TE, Lee CA, Martel MM, Axelrad ME. ODD Symptom Network during Preschool. Journal of Abnormal Child Psychology 2017 May;45(4):743–748. PMID:27523818

19. Bringmann LF, Elmer T, Epskamp S, Krause RW, Schoch D, Wichers M, Wigman JTW, Snippe E. What do centrality measures measure in psychological networks? Journal of Abnormal Psychology 2019 Nov;128(8):892–903. PMID:31318245

20. Richetin J, Preti E, Costantini G, Panfilis CD. The centrality of affective instability and identity in Borderline Personality Disorder: Evidence from network analysis. PLOS ONE Public Library of Science; 2017 Oct 17;12(10):e0186695. [doi: 10.1371/journal.pone.0186695]

21. Bringmann LF, Vissers N, Wichers M, Geschwind N, Kuppens P, Peeters F, Borsboom D, Tuerlinckx F. A Network Approach to Psychopathology: New Insights into Clinical Longitudinal Data. PLOS ONE Public Library of Science; 2013 Apr 4;8(4):e60188. [doi: 10.1371/journal.pone.0060188]

22. Robinaugh DJ, Millner AJ, McNally RJ. Identifying highly influential nodes in the complicated grief network. Journal of Abnormal Psychology 2016;125(6):747–757. [doi: 10.1037/abn0000181]

23. Fried EI, Epskamp S, Nesse RM, Tuerlinckx F, Borsboom D. What are “good” depression symptoms? Comparing the centrality of DSM and non-DSM symptoms of depression in a network analysis. Journal of Affective Disorders 2016 Jan;189:314–320. [doi: 10.1016/j.jad.2015.09.005]

24. Epskamp S, Cramer AOJ, Waldorp LJ, Schmittmann VD, Borsboom D. qgraph: Network Visualizations of Relationships in Psychometric Data. Journal of Statistical Software [Internet] 2012 [cited 2020 Feb 13];48(4). [doi: 10.18637/jss.v048.i04]

1. Developer Agreement and Policy are available on: <https://developer.twitter.com/en/developer-terms/agreement-and-policy>. [↑](#footnote-ref-1)
